# Risk Factors for Methicillin-Resistant *Staphylococcus aureus* Carriers in the Intensive Care Unit: A Single-Center, Retrospective Cohort Study in Japan

**DOI:** 10.1155/cjid/5747507

**Published:** 2025-07-28

**Authors:** Hisato Yoshida, Masayuki Nigo, Kyoko Hisada, Takahiro Tokunaga, Shinpei Matsuda, Hitoshi Tsukamoto, Koji Hosokawa, Ippei Sakamaki, Hitoshi Yoshimura, Hiromichi Iwasaki

**Affiliations:** ^1^Department of Dentistry and Oral Surgery, Unit of Sensory and Locomotor Medicine, Division of Medicine, Faculty of Medical Sciences, University of Fukui, Fukui, Japan; ^2^Division of Infectious Diseases, Department of Internal Medicine, Houston Methodist Hospital, Houston, Texas, USA; ^3^The School of Biomedical Informatics, University of Texas Health Science Center at Houston, Houston, Texas, USA; ^4^Department of Infectious Diseases, Faculty of Medical Sciences, University of Fukui, Fukui, Japan; ^5^Department of Clinical Laboratory, University of Fukui Hospital, Fukui, Japan; ^6^Medical Research Support Center, University of Fukui Hospital, Fukui, Japan; ^7^Research Promotion Office, Shinseikai Toyama Hospital, Toyama, Japan; ^8^Department of Pharmacy, University of Fukui Hospital, Fukui, Japan; ^9^Department of Anesthesiology and Reanimatology, Faculty of Medical Sciences, University of Fukui, Fukui, Japan; ^10^Division of Infection Control, University of Fukui Hospital, Fukui, Japan

**Keywords:** active surveillance cultures, intensive care unit, methicillin-resistant *Staphylococcus aureus*, methicillin-sensitive *Staphylococcus aureus*

## Abstract

**Background:** Methicillin-resistant *Staphylococcus aureus* (MRSA) is a common pathogen in the intensive care unit (ICU). Active surveillance cultures (ASCs) for MRSA are often performed in ICUs; however, they may not be optimal in ICUs with a low MRSA prevalence. This study aims to determine the risk factors of MRSA carriage in the ICU and develop a clinical predictive model to optimize the screening process.

**Methods:** All patients who were admitted to the ICU between April 2015 and August 2022 were retrospectively included in this study. At the time of ICU admission, all patients underwent MRSA screening using nasal ASCs. Based on the screening results, patients were categorized into MRSA-positive and MRSA-negative groups. Patients' characteristics were evaluated to determine the prevalence of MRSA and the risk factors. Cost analysis was conducted based on the risk factors identified by our analysis.

**Results:** Of the 3927 ICU patients included, 133 (3.4%) were MRSA-positive. Multivariate analyses showed that risk factors for MRSA carriage were age ≥ 50 years (odds ratio [OR]: 2.11), history of hospitalization within a year (OR: 1.50), and ICD-10 codes classification I, IV, and XII (OR: 4.98). Screening patients based on at least one of the risk factors exhibited high sensitivity (96.9%) to identifying MRSA carriage and could reduce ASC overall costs by 10.9%, equivalent to $4686.

**Conclusion:** This study suggests that universal ASCs to detect MRSA may not be optimal in ICU settings with a low prevalence of MRSA. Targeted screening based on risk factors may reduce the volume and cost of MRSA screening. Prospective multicenter studies are warranted to validate these findings and to assess the generalizability of the proposed screening strategy.

## 1. Introduction

Patients admitted to the intensive care unit (ICU) are at risk of infection due to various underlying medical conditions, medical devices, and invasive procedures, such as surgery [1]. In the EPIC II study of infections in ICUs in 76 countries, 51% of patients admitted to ICUs were reported to have complications of infection [2]. Similarly, in the Japanese Survey of AntimiCRobial Use in ICU PatienTs (JSCRIPT) study, which investigated the use of antimicrobial agents in ICUs in Japan, 50.1% of patients admitted to ICUs had some kind of bacterial infection, and 72.6% of all patients were treated with intravenous antimicrobial agents [3]. ICU patients are at high risk of harboring drug-resistant bacteria due to antimicrobial exposures and can transmit those bacteria to other patients in ICUs.

Methicillin-resistant *Staphylococcus aureus* (MRSA) is a major human pathogen that causes both community-acquired and nosocomial infections and is a common pathogen in ICUs, causing significant morbidity and mortality [4]. Identification of MRSA carriers is a crucial first step to isolate those patients and prevent transmission of the pathogens in the ICU [5–7]. Active surveillance cultures (ASCs) on specimens collected from relatively easy-to-detect sites are used to identify patients with drug-resistant bacteria in the ICU. For MRSA, ASC from nares is recommended to detect the carriers early and to enhance contact precautions [8]. However, in Japan, there are a few reports on the MRSA carriage and risk factors in patients admitted to the ICU.

Due to limited molecular diagnostic resources in Japan, culture methods are often used as an alternative method. However, the use of ASCs to detect patients with MRSA in the ICU has several limitations. First, while a rapid turnaround time is required to proactively isolate infected patients, ASCs require additional days to identify infected patients compared to polymerase chain reaction (PCR)–based methods, which are widely used in the U.S. and Europe. Second, in settings with a low prevalence of MRSA, such as Japan, cost effectiveness is likely compromised in the screening process. MRSA positivity rates differ between Japan and European countries. In Europe, MRSA positivity in 2015 ranged from about 10% to 34%, depending on the country [9]. In contrast, the prevalence of MRSA in Japan in 2015 was 6.6% [10]. Furthermore, in East Asia, including Japan, the incidence of MRSA has gradually decreased from 2008 to 2015 [11]. Given the international variation in MRSA prevalence, universal screening may not be optimal in low-prevalence ICU settings such as Japan. Identifying high-risk patients and adopting a targeted screening approach may provide a more cost-effective and practical solution.

The aim of this study is to determine the prevalence of MRSA carriers in the ICU at a rural Japanese tertiary care center, identify the risk factors of MRSA carriage in an ICU setting with a low prevalence of MRSA, and evaluate if targeted screening for MRSA carriage could optimize the screening process and reduce the costs.

## 2. Materials and Methods

### 2.1. Study Design and Sample

This was a retrospective, single-center study using the ASC database of the University of Fukui Hospital, Fukui, Japan. All patients admitted to the ICU of the University of Fukui Hospital between April 2015 and August 2022 were included in the study. For patients who entered the ICU more than once during the study period, only ASCs at the first admission were included.

### 2.2. Active Surveillance Protocol for the Detection of MRSA

All patients were screened for MRSA by ASC at the time of ICU admission. Nasal swab specimens were obtained from the patients. Patient samples were incubated at 37°C on two types of agar media: Accurate™ Separated Sheep Blood Agar/BTB Lactose Agar (Shimadzu Diagnostics Co., Ltd., Tokyo, Japan) and Chocolate Agar (Kyokuto Pharmaceutical Industrial Co., Ltd., Tokyo, Japan). Colonies grown on the agar plates were identified as *Staphylococcus aureus* (*S. aureus*) using mass spectrometry (MALDI Biotyper®, Beckman Coulter Inc., Brea, CA, USA). The MALDI Biotyper displays identification scores in a color-coded format: scores ranging from 2.00 to 3.00 are shown in green and indicate high-confidence identification; scores between 1.70 and 1.99 are shown in yellow and indicate low-confidence identification; and scores below 1.70 are shown in red, indicating that no organism identification is possible. In this study, a score of ≥ 2.00 was used as the threshold for reliable species-level identification of *S. aureus*, and isolates with scores below 2.00 were excluded from the analysis. All specimens were incubated for 24 and 48 h without deviation, in accordance with the standardized laboratory protocol. Weekly quality assurance procedures were conducted using *S. aureus* ATCC 25923 as the reference strain for methicillin-susceptible isolates. After identification as *Staphylococcus aureus*, the colonies were inoculated at 37°C on MDRS-K agar medium (Kyokuto Pharmaceutical Industrial Co., Ltd, Tokyo, Japan) and reviewed at 24 h and again at 48 h, if necessary. Yellow-colored colonies appearing at 24 and 48 h were positively identified as MRSA. The culture was considered negative if there were no yellow colonies after 48 h of incubation. The species and methicillin resistance of all positive isolates were confirmed by Microscan WalkAway 96 plus (Beckman Coulter, Inc., Brea, CA, USA).

### 2.3. Study Variables

Demographic data, including age and sex, history of hospitalization within a year, and International Classification of Diseases 10th Revision (ICD-10) codes of ICU admission diagnosis were collected for all patients. ICD-10 codes are a classification system for distinguishing and organizing medically similar diseases, injuries, and conditions [12]. ICD-10 codes I (certain infectious and parasitic diseases), IV (endocrine, nutritional, and metabolic diseases), and XII (diseases of the skin and subcutaneous tissue) were selected based on previous literature showing associations with MRSA infection or colonization [4, 13–16]. These three classifications were dichotomized into a binary variable indicating the presence or absence of any of the codes in the patient's primary ICU admission diagnosis. All available clinical data were retrospectively obtained by reviewing electronic medical records.

### 2.4. Study Endpoints

Patients were categorized as MRSA-positive or MRSA-negative according to MRSA nasal culture results of active surveillance. The primary study endpoint was the rate of MRSA carriage in patients admitted to the ICU. The secondary endpoint was the cost of ASC.

### 2.5. Analysis of Costs for ASC

Using risk factors identified by the logistic regression model, we evaluated the association between the rate of MRSA carriage at ICU admission and the cost of ASC. The cost analysis in this study was conducted over the entire study period, from 2015 to 2022. Briefly, the risk factors for MRSA carriage were combined to calculate the number of MRSA-positive cases and determine the MRSA-positive rate. We then calculated the cost of ASC for each combined risk factor and determined the change in the cost by considering that the following ASC costs were included in the analysis: cost of nasal swab, agar medium, panel for testing antimicrobial susceptibility, and labor cost. The conversion from Japanese yen to U.S. dollars was made at 147 yen to 1 U.S. dollar (January 24, 2024). The cost of ASC per specimen was $0.5 for the nasal swab, $1.7 for the MDRS-K agar medium, and $8.5 for labor, for a total of $10.7. Antimicrobial drug susceptibility testing was performed for MRSA-positive cases, which cost an additional $7.5.

### 2.6. Statistical Analysis

All statistical analyses were performed using IBM SPSS Statistics 29 (IBM, Tokyo, Japan). Associations between categorical variables in MRSA detection were analyzed using the chi-square test or Fisher's exact test, as appropriate. Adjusted residuals for each categorical variable were calculated. For multivariate analysis, variables were selected a priori based on clinical relevance and previous literature, rather than on statistical significance in univariate analysis. These selected variables were entered into a logistic regression model without stepwise elimination. The model's discriminatory performance was assessed using the area under the receiver operating characteristic (ROC) curve (AUC), and model fit was evaluated with the Hosmer–Lemeshow goodness-of-fit test. No formal power calculation was conducted due to the retrospective nature of the study. Continuous variables are presented as mean ± standard deviation (SD). A *p* value < 0.05 was considered statistically significant.

The study protocol was approved by the Ethics Committee of the Faculty of Medical Sciences, University of Fukui (Reference ID: 20230009). The need for informed consent was waived owing to the retrospective nature of the study. As opt-out policy, the information of study was cited on the hospital website and the participants were allowed to deny the inclusion to the study via a direct contact to the researchers.

## 3. Results

**Figure 1:**
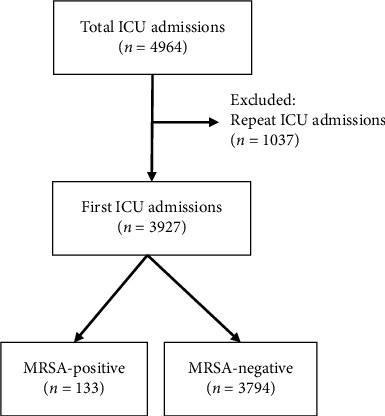
Flowchart of patient inclusion and MRSA screening.

During the study period, a total of 4964 ICU admissions were recorded. Among them, 1037 were repeat ICU admissions. To avoid duplication, only the first ICU admission for each patient was included, resulting in a final cohort of 3927 unique patients (Figure 1). Of these patients, 648 (16.5%) had carriage of *S. aureus* and 133 (3.4%) had carriage of MRSA in the nares. All MRSA-positive cases were susceptible to vancomycin. Characteristics of MRSA carriers at ICU admission are shown in Table 1. The rates of MRSA carriage in the nasal cavity were significantly higher in patients who were ≥ 50 years of age at the time of ICU admission (*p* < 0.05) and in patients who had been hospitalized within the past year (*p* < 0.05). There was a significantly greater rate of MRSA carriage in the group of ICD-10 codes I (certain infectious and parasitic diseases), IV (endocrine, nutritional, and metabolic diseases), and XII (diseases of the skin and subcutaneous tissue) (*p* < 0.001).

Next, the risk factors associated with MRSA carriage were investigetaed using multivariate logistic regression analysis (Table 2analyses showed that the same risk factors for MRSA carriage as the primary analysis. Age ≥ 50 years at ICU admission was a significant independent risk factor for MRSA carriage (odds ratio [OR]: 2.11; 95% confidence interval [CI]: 1.13−3.96; *p* = 0.020) as was the history of hospitalization within one year (OR: 1.50; 95% CI: 1.05−2.13; *p* = 0.025) and ICD-10 codes classification I (certain infectious and parasitic diseases), IV (endocrine, nutritional, and metabolic diseases), and XII (diseases of the skin and subcutaneous tissue) (OR: 4.98; 95% CI: 2.96−8.37; *p* < 0.001). The discriminatory ability of the logistic regression model was assessed using the AUC (AUC = 0.628), indicating modest predictive performance. Model calibration was acceptable, as evaluated by the Hosmer–Lemeshow test (*p* = 0.480).

The association between the rate of MRSA carriage at ICU admission and the cost of ASC is shown in Table 3. Limiting screening at ICU admission to patients with at least one risk factor (3492 [89.1%]) would have identified 129 (96.9%) of the 133 MRSA carriers. Sensitivity to MRSA carriage was highest when at least one risk factor was included. Performing ASCs on all patients admitted to the ICU cost $43,017. Targeted screening for patients with at least one risk factor for MRSA carriage would result in a cost savings of 10.9% ($38,331/$43,017).

## 4. Discussion

Our study examined the MRSA carriage rate identified by ASCs in patients admitted to the ICU and found that only 3.4% of patients were identified as MRSA-positive. We also evaluated the risk factors for MRSA carriage and a targeted screening strategy based on the risk factors in our ICU. We identified that three simple risk factors can be used for the targeted screening: age ≥ 50 years at ICU admission, history of hospitalization within the past year, and ICD-10 codes I, IV, and XII. Our score achieved a sensitivity of 96.9% to identify those patients and could reduce the volume and cost of MRSA screenings in our ICU (10.9% of the screening cost). Our findings indicate that targeted screening based on risk factors may optimize the screening process for MRSA and save the screening costs in our hospital.

The rate of MRSA carriers among patients admitted to ICUs ranged from 4.1% to 11.1%, depending on the reports and geographical locations [17–21]. In Japan, including our hospital, the rates of MRSA carriage in patients admitted to ICUs were around 5%, which is lower than the rates in the U.S., exceeding 10% [17, 18, 20]. Our study is a large single-center study of MRSA carriers in ICU, with a sample size of nearly 4000 patients. Our study revealed even lower MRSA prevalence rates (3.4%) compared to other previous studies from Osaka and Fukuoka prefectures (6.3% and 5.0%, respectively), large metropolitan cities in Japan [17, 18]. Furthermore, according to the Japan Nosocomial Infections Surveillance (JANIS) Annual Open Report 2023, MRSA was isolated from 167,858 inpatients in 2019 (6.02%), 176,848 in 2022 (6.41%), and 183,743 in 2023 (5.95%) among approximately 2.7 to 3 million specimen-submitting inpatients each year [22]. These national surveillance data indicate that the MRSA isolation rate in Japan has remained relatively stable in recent years, underscoring the continued relevance and potential utility of targeted screening strategies. Considering the effectiveness of screenings is significantly affected by the underlying prevalence of diseases and universal screening for MRSA on all ICU admitting patients is costly and time consuming, more flexible approaches are likely necessary, especially in ICUs with a low prevalence of MRSA.

To evaluate potential alternative approaches, we developed a simple risk score for MRSA colonization in our ICU. MRSA carriage in patients admitted to ICU was associated with age ≥ 50 years at ICU admission, history of hospitalization within the past year, and ICD-10 codes I (certain infectious and parasitic diseases), IV (endocrine, nutritional, and metabolic diseases), and XII (diseases of the skin and subcutaneous tissue). These factors can be used to predict MRSA carriage at ICU admission. They can be easily assessed by physicians, nurses, and other medical staff at the time of ICU admission and can contribute to optimizing early isolation strategies to prevent the transmission of MRSA in ICUs. Because this information is readily available and based on ICD-10 codes, the proposed risk score could be easily integrated into clinical workflows. However, successful implementation may still require staff education and incorporation into existing admission protocols or electronic health record (EHR) systems. Using ICD-10 codes allows our score to be generalizable to other institutions with a low prevalence of MRSA because ICD-10 codes are widely used in Japan and other countries [12]. Although these risk factors were significantly associated with MRSA carriage in our cohort, they are not specific to MRSA and may also be observed in other types of drug-resistant bacterial infections. In this study, ICD-10 codes classification I, IV, and XII were identified as risk for MRSA carriage at ICU admission. ICD-10 codes classification I and XII include sepsis-associated and skin and soft tissue-associated infections, respectively, and MRSA infections are likely to be related to these diseases [4, 13–15]. ICD-10 codes classification IV includes diabetes-related diseases, and diabetes mellitus is often associated to MRSA infection or carriage [16]. We chose to use a simple score in this study because it is easy to implement and has high generalizability. In contrast, more complex models using the broad range of data available in EHR, such as machine learning models, can be applied to predict MRSA carriers in the future. Nigo et al. established a deep learning model to predict MRSA culture-positive cases using EHR data [23]. Lin et al. used machine learning to accurately predict which densely populated areas have the highest and lowest risk of MRSA infection over a 14-year span [24]. Achieving higher sensitivity and specificity of the models may lead to larger cost saving compared to the simple scores. However, applying those machine learning models requires additional studies and resources to integrate the models to EHR and clinical workflows.

Our study has several limitations. First, as a retrospective and single-center study, the number of variables investigated was limited, and clinical outcomes related to MRSA carriage were not assessed. Second, nasal cultures were used as the surveillance method, but the culture method is less sensitive than PCR-based screening [25]. In Japan, culture-based MRSA surveillance is commonly performed due to limited access to PCR methods. Culture methods are cheaper and widely available even in rural areas in Japan. Our approach of risk factor–driven targeted screening may provide an additional cost benefit in hospitals using PCR-based surveillance. Third, although our proposed risk score is based on widely used ICD-10 codes and easily obtainable patient characteristics, differences in patient demographics, MRSA prevalence, ICU admission criteria, and infection control policies across institutions or countries may affect its generalizability. Future multicenter studies are needed to validate these findings in broader settings. Finally, this studyutilized nasal swabs alone for MRSA screening at ICU admission, which may havelimited the sensitivity of detection. Although the anterior nares are commonlyused due to their accessibility and relatively high sensitivity (ranging from48% to 93%), MRSA colonization can also occur at other anatomical sites such asthe throat, groin, perineum, wounds, and medical device exit sites.Several studies have demonstrated that additional sampling from sites such asthe throat or perineum increased the sensitivity of surveillance testing toover 90% [26]. Therefore, future studies should consider including cultures from multiple anatomical sites to improve the accuracy of MRSA detection and risk factor assessment.

## 5. Conclusion

We found that the MRSA carriage rate in patients admitted to the ICU was low, and the risk factors for MRSA carriage in patients admitted to ICU were age ≥ 50 years at ICU admission, history of hospitalization within a year, and ICD-10 codes classification I (certain infectious and parasitic diseases), IV (endocrine, nutritional, and metabolic diseases), and XII (diseases of the skin and subcutaneous tissue). Also, screening patients with at least one risk factor for MRSA carriage had high sensitivity in identifying MRSA carriage at the time of ICU admission and could have reduced the number and cost of ASCs. These findings suggest that targeted screenings for MRSA carriage in ICUs with low prevalence of MRSA may optimize the screening process. Prospective studies are warranted to validate our findings and to further develop and refine predictive scoring systems for MRSA carriage in ICUs with low MRSA prevalence.

## Figures and Tables

**Table 1:** Characteristics of MRSA carriers at ICU admission.

	MRSA-positive *N* = 133	MRSA-negative *N* = 3794	*p* value
Age at ICU admission, *n* (%)			
0–49 years	11 (1.8)	614 (98.2)	
≥ 50 years	122 (3.7)	3180 (96.3)	^∗^
Sex, *n* (%)			
Male	83 (3.4)	2392 (96.6)	
Female	50 (3.4)	1402 (96.6)	
History of hospitalization within one year, *n* (%)			
Yes	60 (4.2)	1355 (95.8)	^∗^
No	73 (2.9)	2439 (97.1)	
ICU admission diagnosis, ICD-10 codes, *n* (%)			
I, IV, and XII	18 (12.3)	128 (87.7)	^∗∗∗^
Others	115 (3.0)	3666 (97.0)	

*Note:* After analysis with chi-square test or Fisher's exact test, the adjusted residuals for each categorical variable were calculated. ICD-10: International Classification of Diseases, 10th Revision. ICD-10 codes classification I (certain infectious and parasitic diseases), IV (endocrine, nutritional, and metabolic diseases), and XII (diseases of the skin and subcutaneous tissue).

Abbreviations: ICU, intensive care unit; MRSA, methicillin-resistant *Staphylococcus aureus*.

^∗^ *p* < 0.05.

^∗∗∗^ *p* < 0.001.

**Table 2:** Multivariate logistic regression analysis of risk factors associated with MRSA carriage.

	MRSA detection		*p* value
	Odds ratio	95% CI	
Sex	0.89	0.62–1.28	0.543
Age at ICU admission (≥ 50 years)	2.11	1.13–3.96	0.020
History of hospitalization within one year	1.50	1.05–2.13	0.025
ICD-10 codes classification I, IV, XII	4.98	2.96–8.37	< 0.001

*Note:* Groupings were as follows: ICD-10 codes were divided into classification I (certain infectious and parasitic diseases), IV (endocrine, nutritional, and metabolic diseases), XII (diseases of the skin and subcutaneous tissue), and others. ICD-10: International Classification of Diseases, 10th Revision.

Abbreviations: CI, confidence interval; ICU, intensive care unit; MRSA, methicillin-resistant *Staphylococcus aureus*.

**Table 3:** Utility of various screening strategies and costs of screening by risk factors for MRSA carriage.

Risk factor	Sensitivity (%), *n*	Patients screened (%), *n*	Cost of ASC (U.S. $)	Change in cost (%)
All cases	100 (133/133)	100 (3927/3927)	43,017	
1, 2, or 3	96.9 (129/133)	88.9 (3492/3927)	38,331	10.9
2 or 3	96.2 (128/133)	88.6 (3479/3927)	38,185	11.3
1 or 2	93.2 (124/133)	84.9 (3334/3927)	36,603	14.9
1 or 3	54.1 (72/133)	40.1 (1575/3927)	17,392	59.6

*Note:* Risk factors: (1) ICD-10 codes classification I (certain infectious and parasitic diseases), IV (endocrine, nutritional, and metabolic diseases), and XII (diseases of the skin and subcutaneous tissue), (2) age at ICU admission (≥ 50 years), and (3) history of hospitalization within one year.

Abbreviations: ASC, active surveillance culture; ICU, intensive care unit; MRSA, methicillin-resistant *Staphylococcus aureus*.

## Data Availability

The data used to support the findings of this study have not been made available because of institutional ethics committee permission.

## Nomenclature

ICU: Intensive care unit
MRSA: Methicillin-resistant *Staphylococcus aureus*
ASCs: Active surveillance cultures
*S. aureus*: *Staphylococcus aureus*
MSSA: Methicillin-sensitive *Staphylococcus aureus*
ICD-10: International Classification of Diseases, 10th Revision
